# Development and Evaluations of Transdermally Delivered Luteolin Loaded Cationic Nanoemulsion: In Vitro and Ex Vivo Evaluations

**DOI:** 10.3390/pharmaceutics13081218

**Published:** 2021-08-07

**Authors:** Mohammad A. Altamimi, Afzal Hussain, Sultan Alshehri, Syed Sarim Imam, Usamah Abdulrahman Alnemer

**Affiliations:** Department of Pharmaceutics, College of Pharmacy, King Saud University, Riyadh 11451, Saudi Arabia; salshehri1@ksu.edu.sa (S.A.); simam@ksu.edu.sa (S.S.I.); 441106115@student.ksu.edu.sa (U.A.A.)

**Keywords:** luteolin, breast cancer, cationic nanoemulsion, transdermal delivery, ex vivo permeation parameters

## Abstract

Introduction: Luteolin (LUT) is natural flavonoid with multiple therapeutic potentials and is explored for transdermal delivery using a nanocarrier system. LUT loaded cationic nanoemulsions (CNE1–CNE9) using bergamot oil (BO) were developed, optimized, and characterized in terms of in vitro and ex vivo parameters for improved permeation. Materials and methods: The solubility study of LUT was carried out in selected excipients, namely BO, cremophor EL (CEL as surfactant), labrasol (LAB), and oleylamine (OA as cationic charge inducer). Formulations were characterized with globular size, polydispersity index (PDI), zeta potential, pH, and thermodynamic stability studies. The optimized formulation (CNE4) was selected for comparative investigations (% transmittance as %T, morphology, chemical compatibility, drug content, in vitro % drug release, ex vivo skin permeation, and drug deposition, DD) against ANE4 (anionic nanoemulsion for comparison) and drug suspension (DS). Results: Formulations such as CNE1–CNE9 and ANE4 (except CNE6 and CNE8) were found to be stable. The optimized CNE4 based on the lowest value of globular size (112 nm), minimum PDI (0.15), and optimum zeta potential (+26 mV) was selected for comparative assessment against ANE4 and DS. The %T values of CNE1–CNE9 were found to be ˃95% and CEL content slightly improved the %T value. The spherical CNE4 was compatible with excipients and showed % total drug content in the range of 97.9–99.7%. In vitro drug release values from CNE4 and ANE4 were significantly higher than DS. Moreover, permeation flux (138.82 ± 8.4 µg/cm^2^·h), enhancement ratio (8.23), and DD (10.98%) were remarkably higher than DS. Thus, ex vivo parameters were relatively high as compared to DS which may be attributed to nanonization, surfactant-mediated reversible changes in skin lipid matrix, and electrostatic interaction of nanoglobules with the cellular surface. Conclusion: Transdermal delivery of LUT can be a suitable alternative to oral drug delivery for augmented skin permeation and drug deposition.

## 1. Introduction

Breast cancer is considered as the world’s most prevalent cancer, causing 685,000 deaths with 2.3 million women diagnosed in 2020 [1]. In general, death in women occurs due to metastasis of breast cancer and failure of early detection. However, radiation therapy, surgery, and chemotherapy are applied approaches in the current scenario in healthcare systems. Several synthetic, semisynthetic, and natural compounds have been reported to have potential anticancer activity. However, natural compounds possessing anticancer potential are anticipated to be safer and more compatible compared to synthetic drugs. Commercially available synthetic drugs are associated with several side effects, expensive treatment, and have low compliance in patients.

Luteolin (LUT) (LogP ~ 2.53) is a naturally occurring 2-(3,4-Dihydroxyphenyl)-5,7-dihydroxy-4H-1-benzopyran-4-one possessing potential anti-inflammatory, antioxidation, antimicrobial, antimutagen, apoptosis-inducing, and strong chemo-preventive abilities [2,3,4,5]. The drug is practically insoluble in water (~0.0055 mg/mL) and possesses high lipophilicity (LogP = 2.53) [6]. The drug is a conjugate acid of 2-(3,4-dihydroxyphenyl)-5-hydroxy-4-oxo-4H-chromen-7-olate luteolin-7-olate(1-) with a pka value of 6.5 and molecular weight of 286.24 g/mole. Poor aqueous solubility, instability in the gastric lumen, and low oral bioavailability have limited its clinical application for oral delivery in a conventional dosage form [7,8]. The drug has been reported to have low oral bioavailability (<30%) in a rat model [9,10]. Several reports have been published to improve the solubility, efficacy, and systemic availability of the drug by tailoring it as a nanoemulsion, liposome, self-nanoemulsifying drug delivery system (SNEDDS), solid dispersion, solid lipid nanoparticles (SLNs), and nanostructured lipid carriers (NLCs) [8,11,12,13,14,15].

In this context, LUT may be a suitable candidate for transdermal delivery to control breast cancer when directly applied to the affected area. The approach may be advantageous over conventional oral or parenteral delivery. This method can bypass the first pass hepatic metabolism, avoid gastric instability, have a reduced dose, involve less exposure of other body tissues, and target delivery to the tumor lesion by topical application. Shin et al. investigated follicular delivery of LUT loaded nanoemulsion (oil in water) composed of 5% *w*/*w* poly(ethylene oxide)-block-poly(ε-caprolactone), 20% *w*/*w* sweet almond oil, 1% *w*/*w* lecithin (as emulsifier), and 80% *w*/*w* water as the continuous phase where 3.5 Mm LUT was dissolved in tetrahydro furan (THF) at 45 °C. Furthermore, they found that hair growth was comparable to the drug solution dissolved in organic solvent [14]. In 2020, Ansari et al. explored LUT loaded SNEDDS formulations to improve LUT solubility and permeation across the rat intestinal barrier using castor oil as oil, kolliphor as emulsifier, and polyethylene glycol 200 (PEG 200) as co-emulsifier. They achieved 83- and 17-fold increments in the drug solubility and ex vivo permeation, respectively [12].

Bergamot oil (BO) is a well-known plant-derived essential oil obtained from the mesocarp of *Citrus bergamia* (Rutaceae). This contains 93–96% monoterpenes as major constituents and it has been used as an antiseptic, antifungal, antimicrobial, anthelminthic, analgesic, and anxiolytic, and it facilitates wound healing [16]. Recently, its potential antitumor activity on human SH-SY5Y neuroblastoma cells was studied and it significantly reduced viable cells at a low concentration (0.02–0.03%) by inducing cell apoptosis, mitochondrial dysfunction, and deoxyribose nucleic acid (DNA) fragmentation [17]. Notably, it was found that the combination of d-limonene and linalyl acetate (major components of BO) is able to reduce SH-SY5Y neuroblastoma cells’ viability whereas d-limonene alone did not show antitumor activity [18,19]. Furthermore, poorly water-soluble BO was formulated in a liposomal product for efficient in vivo performance against neuroblastoma cells [19]. BO-derived constituents like limonene, limonene-related monoterpenes, perillyl alcohol, and perillic acid have exhibited potential antiproliferation effects on breast cancer cells for chemotherapeutic applications [20]. Very recently, two potential compounds (brutieridin, and melitidin), derived from bergamot fruit, demonstrated arresting MCF7 cells in the G_0_/G_1_ phase of the cell cycle [21].

LUT has effectively shown potential efficacy against breast cancer by blocking IGF-1-stimulated MCF-7 cell proliferation in a dose- and time-dependent manner, reducing cell viability of MCF-7 and MDA-MB 231-1833, and suppression of the epidermal growth factor receptor signaling pathway followed by antiproliferation of ERα-positive MCF-7 [22,23,24]. In an epidemiological survey, dietary intake (2 mg/dL) of LUT did not show an anticancer effect, which may have been due to the low concentration, and the therapeutic dose may be 10–30 mg/mL [25]. Epithelium mesenchymal transition (EMT) is a key factor to control metastasis and polo-like-kinase 1 (PLK1 as mitotic kinase) regulates G2/M transition for over-expression in cancer metastasis. LUT is able to reverse EMT of MDA-MB-231- and BT5-49-mediated breast cancer cells and inhibits PLK1 gene expression in MCF-7 breast cancer [26,27]. LUT may augment the impact of anticancer drugs to control breast cancer by reducing drug resistance (tamoxifen by inhibiting cyclin E2 expression), promoting apoptosis (by blocking STAT3), and inhibiting breast cancer cell growth [28,29]. Thus, the drug has been found to have potential anticancer activity against breast cancer via diverse mechanistic molecular pathways.

A nanoemulsion is a nanocarrier used for successful transdermal delivery of various lipophilic drugs. This carrier has been found to improve drug solubility and permeation across the main barrier (stratum corneum) of the skin. Particularly, a cationic nanoemulsion for transdermal delivery of LUT using BO possessing innate antitumor potential has not been reported to control breast cancer [20,21]. Therefore, the present study aimed to prepare and evaluate cationic nanoemulsion for transdermal delivery of LUT for improved permeation across rat skin to control breast cancer [30]. In this study, cationic nanoemulsions were developed, optimized, and evaluated for globular size and size distribution, zeta potential, morphology, compatibility, drug content, in vitro drug release, and ex vivo permeation parameters such as permeation flux, drug deposition, and permeation coefficient. Drug release pattern and ex vivo permeation parameters of the optimized formulation were compared with an anionic nanoemulsion (ANE4) and DS (control).

## 2. Materials and Methods

### 2.1. Materials

Luteolin (LUT ˃ 98% purity) was procured from Beijing Mesochem Technology Co. Pvt. Ltd., Beijing, China. Labrasol (LAB) and cremophor EL were obtained from Gatteffosse (36 chem de Genas-BP 603-F-69804 Saint Priest Cedex France) and BASF Cop., (Ludwigshafen, Germany), respectively. Oleylamine (positive charge inducer) was purchased from Sichun Benepure Pharmaceutical Co., Ltd. Sichuan, China. Bergamot oil (BO) was procured from Alpha Chemika, India. Dimethyl sulfoxide (DMSO), sodium hydroxide, and phosphate buffer were obtained from Sigma-Aldrich (St. Louis, USA). Millipore water was used as an aqueous medium.

### 2.2. Methods

#### 2.2.1. Screening of Excipients and Preparation of Cationic Nanoemulsions

##### Solubility Assessment of LUT

LUT is a practically insoluble in water. Therefore, it was required to screen for a suitable oil, surfactant, and co-surfactant to identify the best excipient for nanoemulsion formulation. A weighed amount of LUT was sequentially added to a clear glass vial containing 5 mL of excipient. The vial was kept in a shaker water bath previously set at 40 ± 1 °C for 72 h [31]. Addition of LUT was continued till saturation and an equilibrium was achieved between the dissolved and undissolved drug. This procedure was carried out for each excipient individually under similar experimental conditions. After 72 h, each glass vial containing sample was removed, centrifuged, and the clear supernatant was taken out for analysis. The obtained clear supernatant was diluted with methanol prior to estimation using a UV–Vis spectrophotometer at λ_max_ 350 nm (UV 1601PC, Shimadzu, Tokyo, Japan). The study was replicated to obtain the mean and standard deviation (*n* = 3).

##### Preparation of LUT Loaded Cationic Nanoemulsion and Phase Diagram Construction

The solubility study was performed to select a suitable oil, surfactant, and co-surfactant. Therefore, BO, cremophor EL (CEL), and labrasol (LAB) were selected as an oil, surfactant, and co-surfactant for preparing nanoemulsions. BO and peppermint oil exhibited higher drug solubility. BO was selected due to possessing substantial innate anticancer potential [17]. A constant amount of the drug (30 mg) was dissolved in 30 mg of DMSO to obtain a drug solution. This drug solution was added to BO to obtain a homogenous organic phase containing LUT and positive charge inducer OA (0.5%). CEL and LAB were separately blended in different ratios to obtain various S_mix_ (CEL to LAB ratio) ratios. In general, an oil in water nanoemulsion is achieved by selecting the right combination of surfactant and co-surfactant possessing an HLB value ˃11. Therefore, LAB was added in each S_mix_ ratio. Various pseudo-ternary phase diagrams (PTDs) were constructed by slow emulsification and an aqueous phase titration method [32,33]. Several formulations were formed when the oil–S_mix_ was titrated with aqueous phase at each S_mix_ ratio to delineate a maximum zone of nanoemulsion without phase separation [33]. A nanoemulsion with a maximum delineated region at a minimum consumption of S_mix_ as a prime consideration for safety was preferred. Various S_mix_ values were proportioned in desired ratios (1:1, 1:2, 1:3, 2:1, 3:1, 1:4, 4:1, 2:3, and 3:2) to delineate a precise boundary of the PTD and a stable formulation at room temperature. In brief, each S_mix_ ratio was transferred to the oil phase followed by vigorous mixing to achieve an isotropic blend of pre-concentrate. Then, the obtained pre-concentrate was slowly titrated dropwise using aqueous phase to obtain a cationic nanoemulsion. Thus, several nanoemulsions were prepared and examined for benchtop stability (24 h). Finally, these formulations were characterized for globular size, PDI, and zeta potential before final selection of the most optimized cationic nanoemulsion. These PTDs were generated using Pro-origin 6.0 software (Microcal Software Inc., Northampton, MA, USA). Each formulation contained 3% *w*/*w* of LUT (30 mg per g of cationic nanoemulsion).

#### 2.2.2. Evaluations of Cationic Nanoemulsions

Cationic nanoemulsions (CNE1–CNE9) were expected to be thermodynamically stable with dispersed nanoscale globules with size and size distribution within acceptable ranges. The size and size distribution (PDI) were measured using a Zetasizer Nano ZS working on the principle of “dynamic light scattering” (Malvern Instruments, Worcestershire, UK). The DLS principle of this technique was non-invasive and the particles were measured under constant Brownian motion (random thermal diffusion motion). Particles diffuse at a speed relative to their size, and Brownian motion varies under temperature. Therefore, measurements was taken at a constant temperature for a precise assessment of globular size. The sample was diluted with distilled water (100 times) before analysis. Each sample was measured at an operating temperature of 25 °C and a scattering angle of 90° [34]. Similarly, zeta potential was measured under same experimental conditions without dilution. The study was replicated to obtain the mean and standard deviation (*n* = 3). Zeta potential is the surface charge imposed on particles already dispersed in a given medium. Theoretically, the values of zeta potential may be zero, positive, or negative. In this study, OA was imposed over the globular surface for the expected positive zeta potential. The sample was processed using a Zetasizer coupled with a 4.0 mW He-Ne red laser (633 nm) able to measure zeta potential values in the range of ± 120 mV [34]. Final pH was measured using a digital HI 9321 pH meter (Hanna Instruments, Ann Arbor, MI, USA).

#### 2.2.3. Freeze–Thaw Cycles and Centrifugation Tests

A nanoemulsion is a transparent system containing globules with a mean diameter size range of around 100 nm and it is considered as a kinetically stable system [35]. Therefore, it was required to assess thermodynamic stability of the developed cationic nanoemulsions (CNE1–CNE9 and ANE4) at varied temperatures and under stressful centrifugal force. For this, alternative freezing and thaw cycles were repeated for freshly prepared nanoemulsions. Therefore, each formulation was stored in a clear glass vial and placed in a freezer at (−18 °C) for 18 h. Then, the sample was removed and placed at room temperature (~23 °C) for 18 h to return to the original state. Then, the formulation was sent back to freezer at the same temperature for a further 18 h to complete the second cycle. Similarly, the same formulation was kept in an oven maintained at 40 °C for 2 h, following the same procedure. Freezing and thawing cycles were repeated for three cycles and observed for any signs of physical instability (phase separation and drug precipitation) [36]. In order to test the ability to withstand mechanical stress, each formulation was subjected to centrifugation (Aat 10,000 rpm for 5 min. The experiment was replicated three times (*n* = 3) for any signs of physical instability.

#### 2.2.4. Percent Transmittance

For this, the samples (CNE1–CNE9) were transferred to a UV cuvette and the percent transmittance value was measured using a UV–Vis spectrophotometer. The sample (1 mL) was diluted (100 times) with Millipore water before analysis. Percent transmittance of each sample was estimated at 210 nm against Millipore water as a blank [37].

#### 2.2.5. Morphological Assessment

Transmission electron microscopy (TEM) is the most sophisticated and advanced technology to measure morphological properties of nanoscale particles. It measures the particle size (diameter) and shape dispersed in aqueous phase. The samples were gently poured over a copper grid followed by carbon coating. The sample (CNE4) was negatively stained with phosphotungstic acid (0.1% *w*/*v*) and scanned with TEM (JOEL, 120 KV, FEI Company, Tokyo, Japan). Before analysis, the sample was completely dried for simplified visualization under varied magnification. Notably, the size measured with TEM is slightly different from the size measured with the DLS technique. This is due to instrumental error. This type of variation is expected due to differential adsorption of particles or globules after placing them on a copper grid. Therefore, this difference is expressed as the fold error (FE) [31]. This variation is estimated using Equation (1):

Fold error = 10^Log (particle size, TEM/particle size, zetasizer)^ (1)

Z-average mean size (D_z_) = [(Σ *S_i_*)/Σ (*S_i_*/*D_i_*)] (2)

where D_z_ represents the hydrodynamic diameter (intensity-based harmonic mean) of the globular particle. *S_i_* and *D_i_* indicate the scattered intensity from the particle *i* and the diameter of the particle *i*, respectively.

#### 2.2.6. Chemical Compatibility Study: FTIR Study

In order to negate any chemical incompatibility, an attenuated total reflection–Fourier transform infrared (ATR-FTIR) study was carried out for BO, CEL, LAB, blank CNE4, pure LUT, and LUT loaded LCNE4. The sample was smeared on the sample holder for processing using ATR-FTIR spectroscopy (Bruker Alpha, Ettlingen, Germany). The method is sensitive, fast, non-destructive, and highly reproducible. Spectra were normalized and the baseline was corrected using OPUS software.

#### 2.2.7. Drug Content

The drug contents of all developed formulations were determined using 1 mL of the sample (from each nanoemulsion) and the sample was dissolved completely in methanol (10 mL). Then, the mixture (in a tightly closed glass vial) was placed in a shaker under constant stirring maintained at 37 ± 1 °C for 2 h. Later, the supernatant was used to estimate the drug content using a spectrophotometer [37]. The drug was analyzed by taking absorbance at 350 nm against methanol as a blank. The analysis was replicated to obtain the mean and standard deviation (*n* = 3).

#### 2.2.8. Drug Release Study

The drug suspension (DS), CNE4, and ANE4 were used to investigate in vitro release pattern in phosphate buffer medium (pH 6.8) using a dialysis membrane. Each formulation containing LUT (30 mg/mL) was loaded in a dialysis membrane (Himedia, Ltd., Mumbai, India, molecular weight cut-off of 12–14 kDal) previously activated in the release medium for 12 h [8]. The dialysis membrane was tied on both sides and immersed in the release medium (500 mL). The release medium was under constant stirring at 100 rpm using a Teflon coated magnetic bead, and the medium was maintained at a constant temperature (37 ± 1 °C). Sampling (1 mL) was performed at varied time points (1, 2, 4, 6, 8, 10, and 12 h) and the sample was analyzed using a spectrophotometer at 350 nm. Notably, the withdrawn sample volume was replaced with fresh medium to maintain sink conditions. The test sample (1 mL withdrawn) was first filtered using a membrane filter, and then analyzed. The release mechanism was evaluated by applying several mathematical models (zero order, first order, and Higuchi).

#### 2.2.9. Ex Vivo Permeation Studies

The permeation parameters of CNE4, ANE4, and DS were investigated using rat skin. These parameters were cumulative amount permeated, permeation flux (*f*), and enhancement ratio (ER) of the formulations intended for transdermal delivery. Targeted flux was calculated based on the values in the literature. Wistar rats (weighing about 250–350 g and 6–8 months old) were approved by the Institutional Animal House (Institutional Ethical Committee), College of Pharmacy, King Saud University, Riyadh (approval number KSU-SE-20-64) [31]. Rats were caged properly with free access to food and water. Animals were acclimatized for 12 h before the experimental procedure. The protocol was followed as per ARRIVE guidelines.

Initially, rats were ethically sacrificed. Hair and fatty debris were removed from the abdominal skin using surgical scissors. Franz diffusion cells were used for the permeation study. The processed skin was placed between both chambers (receptor and donor) so that the epidermis portion faced the donor chamber for the sample loading. The receptor chamber was filled with 22.5 mL of PBS (pH 7.4) and maintained under constant stirring (100 rpm) using Teflon coated magnetic rice beads [38]. The acceptor medium was maintained at 32 ± 1 °C throughout the study. The sample (0.33 mL containing 9.9 mg of luteolin) was loaded into the epidermal portion of the donor chamber. Sampling was carried out at varied time points (1, 2, 3, 6, 12, 20, and 24 h). The collected sample was pre-filtered (membrane filter) and the drug was quantified at 350 nm using a validated HPLC method (Waters, MA, USA) equipped with a reverse phase C18 column (Waters, SunFire^®^, 5 µm) (150 mm× 4.6 mm, 5 µm particle size of stationary packing in column) and a binary pump (Waters 1525, USA). The DS (10 mg/mL) served as a control. The adhered sample from the skin was removed by washing with PBS after 24 h. The drug was quantified using mobile phase composed of acetonitrile (ACN), methanol, and water (containing 1% *v*/*v* acetic acid buffer at pH 4) in the ratio of 60%:30%:10% *v*/*v*/*v*. The mobile phase was filtered using a 0.45 µm membrane filter to remove any suspended particles, followed by bath sonication to avoid gas bubbles. The sample was injected (20 µL) for analysis over a total run time of 5 min at a flow rate of 1.0 mL min^−1^. A standard linear calibration curve for LUT was drawn in methanol with a regression coefficient of *r^2^* ≥ 0.99.

The values of *J_ss_* (steady state flux) were obtained from the linear slope of the cumulative LUT permeated over 24 h. The permeation coefficient (Pc) was obtained from the *J_ss_* and the loaded concentration on the epidermis surface (C) of LUT (Pc = *J_ss_*/C). Notably, targeted flux was estimated using Equation (3) to confirm therapeutic efficacy of CNE4, ANE4, and DS [39].

Targeted flux (*J_t_*) = (C_ss_ × C_t_ × BW)/A (3)

where C_ss_ represents the steady-state concentration of LUT in rat plasma (0.167 µg/mL) to decide the therapeutic window. C_t_ indicates total body clearance (13.996 mL/Kg/h), and BW = standard body weight of the investigated rat (0.25–0.3 Kg). The value of “A” is the skin effective area used to apply formulations for diffusion levels across skin (=2.34 cm^2^).

A roughly calculated value range of *J_t_* for LUT was 0.24–0.299 µgcm^−2^h^−1^ as the therapeutic window based on the values from the literature [8]. The calculated value range of targeted flux was a rough estimation of the LUT concentration expected to be fluxed in the plasma after topical application of the investigated nanocarrier. However, it is a well-known fact that several physiological and physicochemical properties of the drug, as well as the nanocarrier, have a significant impact on the permeation parameters. It was reported that an improved transdermal diffusion rate (using rat skin as a dynamic ex vivo model) facilitates enhanced percutaneous permeation, and the ex vitro model is static [40]. Moreover, the permeation rate was expected to be even higher in in vivo conditions.

Finally, drug deposition (DD) was studied after completion of the ex vivo permeation study. The skin sample was removed from the Franz diffusion cell along with the sample. The adhered sample was removed carefully using running water. The exposed skin area (effective area responsible for permeation during the experiment) was properly excised from the skin portion and excess skin was removed using surgical scissors. The obtained skin was then sliced into small pieces and placed in a beaker containing equal volumes of methanol and chloroform (10 mL). The mixture was stirred for 12 h using a magnetic stirrer at 37 °C. Finally, the mixture was centrifuged to separate out tissue debris, and the supernatant was used for LUT estimation. The extracted drug content was quantified using HPLC at 350 nm.

## 3. Analysis Method

The drug analysis was carried out using a validated high-performance liquid chromatography (HPLC) method. In brief, the drug was assayed in in vitro and ex vivo studies using a reverse C18 column (150 mm × 4.5 mm, 5 µm as particle size of packing material). Analysis was carried out at room temperature (25 °C) in a replicated manner (*n* = 3). The mobile phase was composed of acetonitrile (60%), methanol (30%), and 10% water (containing 1% acetic acid, *v*/*v*). The final pH was set at 4.0 for maximum stability and drug solubilization. The mobile phase was freshly prepared, filtered (using a membrane filter), and subjected to bath sonication to remove dissolved gases. The analysis was performed in isocratic mode with a flow rate of 1 min/mL and injection volume of 20 µL. The complete chromatogram was obtained over a run time of 8 min. The drug was analyzed using a UV detector at an absorption wavelength of 350 nm [7,13]. A standard calibration curve was obtained over a range of 20.0–100 µg/mL with a regression coefficient correlation (r^2^) of 0.99. The values of the lower limit of detection (LLOD) and lower limit of quantifications were found to be in the range of 0.2–1.0 µg/mL and 0.5–2.0 µg/mL, respectively, as validation parameters.

## 4. Results and Discussion

### 4.1. Solubility Assessment and Selection of Excipients

**Figure 1:**
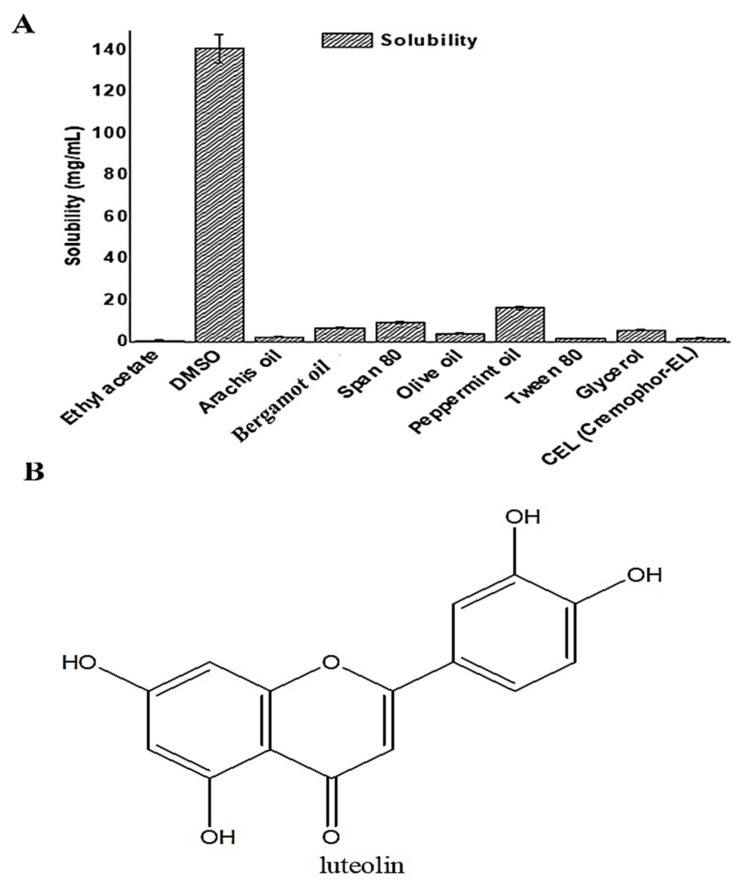
(**A**) Solubility of LUT in various oils, surfactants, and co-surfactants, and (**B**) chemical structure of luteolin.

LUT is a poorly soluble drug in water. Therefore, it was important to identify a suitable solvent and oil for fabricating nanoemulsions. Peng et al. reported the aqueous solubility of LUT as 0.00055 mg/mL at 30 °C which can be a rational selection parameter of suitable excipients for formulation development [6]. The study aimed to formulate a cationic nanoemulsion ferrying LUT for transdermal delivery to control breast cancer, when applied to the affected tumor lesion, and electrostatic-mediated augmented cellular internalization during permeation across the skin’s SC layer. Therefore, it was a prerequisite to find a suitable solvent, surfactant, co-surfactant, and oil to tailor the nanoemulsion with the proper ratio of excipients and stabilized product. The solubility values are presented in Figure 1A. Maximum solubility was obtained in DMSO (141.08 ± 6.98 mg/mL) whereas ethyl acetate showed the minimum solubility (1.09 ± 0.05 mg/mL) among the explored excipients. The solubility values in arachis, BO, olive, and peppermint oils were found to be 2.63 ± 0.13 mg/mL, 6.92 ± 0.35 mg/mL, 4.32 ± 0.22 mg/mL, and 16.57 ± 0.83 mg/mL, respectively. Thus, peppermint exhibited better solubility of LUT among the explored oils. However, BO was selected for formulation development due to it being a well-explored natural oil possessing innate anticancer potential, as mentioned before. This approach may synergize an additive effect in combination with LUT if loaded in a nanoemulsion. OA was used to impose cationic charge on the nanoscale carrier which may facilitate electrostatic interaction-mediated bioadhesion with tissue (negative charge surface) for prolonged drug exposure and subsequent permeation. Therefore, a combination of BO, OA, and DMSO was used for the phase diagram study. The oil is reported to exhibit anticancer activity due to two prime constituents, d-limonene and linaly acetate [18]. Moreover, BO is obtained from a natural source and considered to be safe and biocompatible as compared to semisynthetic lipid. Additionally, BO may elicit synergistic antitumor potential if loaded with LUT (Figure 1B). This approach may offer several benefits, such as (a) reduction in unnecessary introduction of excipients in the patient’s body, (b) synergistic approach may reduce the dose and dose-dependent toxicity, (c) a cost-effective product, and (d) high patient compliance.

### 4.2. Preparation of LUT Loaded Cationic Nanoemulsion and Phase Diagrams

**Figure 2:**
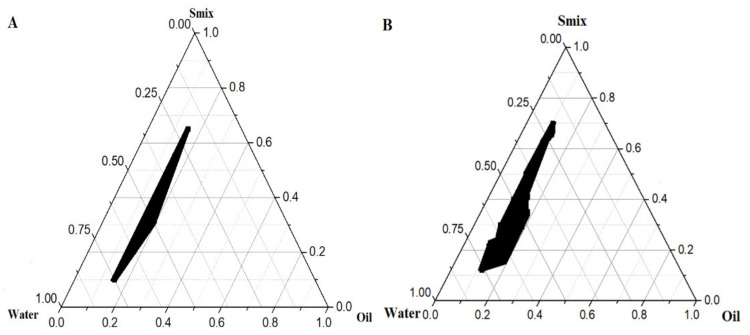
Pseudo-ternary phase diagrams of (**A**) optimized cationic nanoemulsion CNE4, and (**B**) anionic ANE4 containing luteolin (S_mix_ = 2:1).

Several PTDs were constructed using screened BO, CEL, and co-surfactant (LAB). To impose a positive charge on the nanoemulsion, a fixed amount (0.05% *w*/*w*) of cationic charge inducer (OA) was also added to the organic phase [30]. A constant amount of the drug (30 mg) was dissolved in the DMSO–BO mixture and thus the organic phase contained LUT, DMSO, OA, and BO. On the other hand, S_mix_ ratios had varying concentrations of the surfactant to the co-surfactant, and vice versa (1:1, 1:2, 1:3, 1:4, 4:1, 2:1, 3:1, 3:2, and 2:3). In general, surfactant and co-surfactant were selected based on their hydrophilic lipophilic balance (HLB) values (˃10) to achieve a stable oil in water (o/w) nanoemulsion. The HLB values of CEL and LAB are 13 and 14, respectively. Moreover, CEL exhibited comparable solubility (~2.1 mg/mL) of LUT as observed in hydrophilic and viscous Tween 80 (2.09 mg/mL). LAB is also reported to function as an efflux inhibitor and was expected to produce a nanoemulsion with reduced globular size when blended with a surfactant such as CEL [30]. Using the organic phase and various ratios of S_mix_, several PTDs were constructed by a slow titration method with an aqueous phase [33]. We illustrate stable (with no signs of instability) formulations at certain S_mix_ ratios in Figure 2. In this method, incorporation of non-ionic and amphiphilic LAB improved CEL-based emulsification efficiency, decreased the oil–water interfacial surface tension, and is consequently considered as a potential approach to reduce the content of surfactant in S_mix_ [33,41]. Pharmaceutical scientists focused on using heterogeneous non-ionic CEL (polyoxyl 35 castor oil), which may be attributed to its ability to solubilize, emulsify, improve topical absorption, skin permeability, and protection, and encapsulate lipophilic drugs such as the commercialized product paclitaxel (50% cremophor EL) [41]. Moreover, CEL is associated with a lower degree of ethoxylation and unsaturation which can be expected to produce nanoemulsions with smaller sizes and narrow size distributions as compared to viscous cremophor RH40 [41]. In the case of LAB, it is a chemical PEG-8 caprylic/capric glyceride and used as a co-surfactant. Several authors exploited LAB as a co-surfactant or surfactant to tailor stabilized microemulsions for cutaneous delivery of various lipophilic drugs, which may be due to its ability to avoid skin irritation and potential skin permeation effect [42]. In general, the emulsification efficiency of LAB depends upon several factors, such as (a) type of oil, (b) the molecular volume of oil, (c) chemical structure of oil, (d) polarity of oil, (e) the solubilization capacity of the surfactant–oil mixture, (f) physicochemical properties of the surfactant, and oil concentration [42].

### 4.3. Evaluations of Prepared Nanoemulsions

The optimized formulation CNE4 was the most robust cationic nanoemulsion (maximum delineated area in phase diagram with ratio of 2:1) with a suitable globular size (110.6 ± 8.1 nm), PDI (0.15), and zeta potential (approximately +26 mV) at an S_mix_ ratio of 2:1 (Figure 2). The detailed composition of formulations is summarized in Table 1. The positive charge imposed on the globular surface indicates stabilized and substantially emulsified CNE4 ferrying lipophilic LUT. OA is a hydrophobic compound and has been reported to provide highly monodispersed nanoparticles, which may be attributed its electrostatic repulsion among globules dispersed in the continuous phase [43]. Tsai et al. investigated the significant impact of functionalized PEG-OA used as an amphiphilic surfactant for synthesis of gold nanoparticles for improved epidermal permeation and in vivo efficacy [43]. It was observed that LAB had a substantial impact on globular size (decreased from 373 nm to 158 nm) from CNE1 to CNE3 (1:1 to 1:3) which can be attributed to the relatively increased concentration of LAB in S_mix_, as observed in Table 1. However, zeta potential values were approximately constant. In contrast, on increasing the relative concentration of CEL in S_mix_, the globular size was found to be increased significantly, i.e., 110.6 nm, 307.0 nm, and 407.5 nm in 2:1 (CNE4), 3:1 (CNE5), and 4:1 (CNE7), respectively. The nanoemulsion ANE4 (OA free) exhibited globular size, PDI, and zeta potential of 134.0 nm, 0.171, and −28.9 nm, respectively (Table 1). Thus, the overall ranges of size, PDI, and zeta potential values for the developed formulations (CNE1–CNE9) were found to be 110–407 nm, 0.15–0.82, and +14.6–39.0 mV, respectively.

In this study, the imposed positivity on globular size of the developed nanoemulsions was purposely used to achieve (a) electrostatic interaction with skin cells, (b) augmented colloidal stability due to electrostatic repulsion between them, (c) increased skin permeation across the skin strata due to possible OA-PEG-mediated reversible changes in skin protein layer [43], and (d) reduced chances of Ostwald ripening [43,44]. All formulations were set at physiological pH (~7.4). These developed nanoemulsions were further subjected to a thermo-mechanical stress test (freeze–thaw cycles of thermodynamic stability test with subsequent centrifugation) (Table 2).

### 4.4. Freeze–Thaw Cycles and Centrifugation Tests

In order to test the thermodynamic stability of the developed formulations, it was vital to assess the capability of these formulations to cope with the thermo-mechanical stress tests. Two extreme temperatures (−21 and 40 °C) and intermittent room temperature were used to screen stable formulations. A study reported that LUT was soluble in oil at an elevated temperature and then formed multiple needle-shaped crystals after cooling to a low temperature. This was explained by phase separation occurring due to the π–π transition between the aromatic rings of neighboring chroman-4-one as well as H-bonding between the –OH group and –CO functional group of adjacent LUT. Furthermore, this crystal growth phenomenon with cooling was completely suppressed by formulating LUT loaded nanoemulsions by aiding thermal motion and drop to drop repulsion [14]. In the present study, cationic nanoemulsions were stable under thermal and mechanical stress which may be correlated with the imposed repulsion. Those formulations showing any signs of instability (phase separation, turbidity, nucleation for crystal growth, and precipitation) due to possible metastable formulation were discarded and dropped from further evaluations. Results are presented in Table 2 where CNE6 and CNE8 failed due to greater turbidity and phase separation. This test suggests a long-term shelf-life of nanoemulsions as compared to conventional emulsions [45]. The failed formulations were unable to return to their initial transparency, isotropic behavior, and physical stability. This may be due to the relatively higher values of size and PDI, and the low content of CEL in S_mix_.

### 4.5. Percent Transmittance (%T)

**Figure 3:**
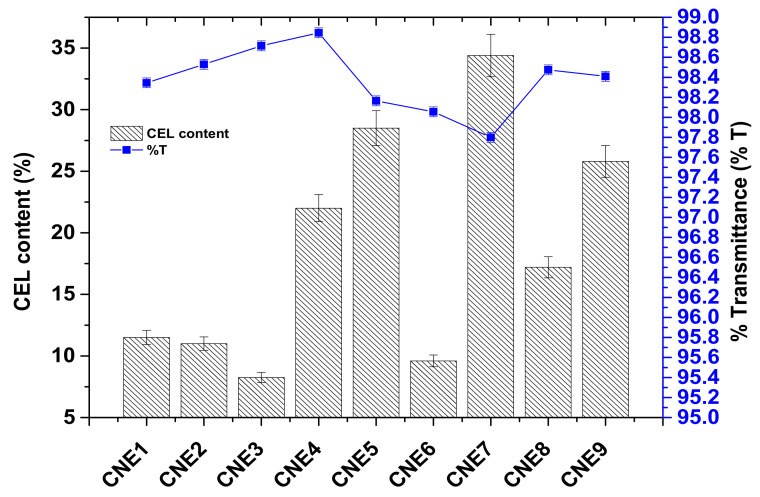
Impact of surfactant (CEL) on %transmittance in various formulations (CNE1–CNE9).

Results of %transmittance obtained from various formulations are shown in Figure 3. These values ranged from 97.8 to 98.8% for all nine formulations. The obtained %T values were found to be invariable and comparable to the water blank, suggesting a transparent and isotropic nature of CNE1–CNE9. Upon close examination of these values, the impact of surfactant “CEL” was observed to be weak from 11.5 to 22.0% *w*/*w* whereas there was a progressive decline in %T till 34.4% of CEL, as shown in Figure 3. In formulations CNE1–CNE9, the concentration of CEL is different due to varied ratios of CEL in the S_mix_ ratio, such as 1:1 (50%), 1:2 (33%), 1:3 (25%), 1:4 (20%), etc. (as shown in Table 1). It is clear that a relatively high content of LAB (as compared to CEL) caused a slight increase in %T. In CNE4, CNE5, and CNE7, with the relative increase in the concentration of CEL, as compared to LAB, the %T value was found to be slightly decreased, suggesting that LAB and CEL functioned as an efficient emulsifying surfactant and co-surfactant, respectively. In the graph, it is clear that there is no significant difference in %T values for the CNE1–CNE9 formulations. Thus, the overall result showed insignificant variation (*p* ˃ 0.05) in %T values over the explored concentration range of CEL in the formulations.

### 4.6. Morphological Assessment

TEM was performed to assess morphological shape, size, and nature of the globular distribution (chance of aggregation and dispersed heterogeneous globules) of the optimized nanoemulsion blank and LUT loaded CNE4. In general, the prepared nanoemulsions were expected to be spherical in morphology, distinctly dispersed due to the imposed cationic charge of the surface, and considerably stable (free from any signs of globular aggregation). The globular size estimated using the TEM technique differs slightly from those obtained from the DLS-based size assessment. This was obvious due to instrumental errors during the sample processing and scanning under an electron beam. A few studies have suggested that this is possibly due to preferential adsorption of relatively smaller globules when placed on the copper grid. Therefore, this variation was expressed as a “fold error” (FE) and was expected to be below 2.0 as an acceptable range (FE ˂ 2.0). The globular size of CNE4 was the same as that obtained with the DLS technique. Moreover, the efficiency of the dermal/epidermal delivery of LUT depends upon the globular size of the cationic nanoemulsion; the smaller the size, the deeper it may be delivered.

### 4.7. Chemical Compatibility

**Figure 4:**
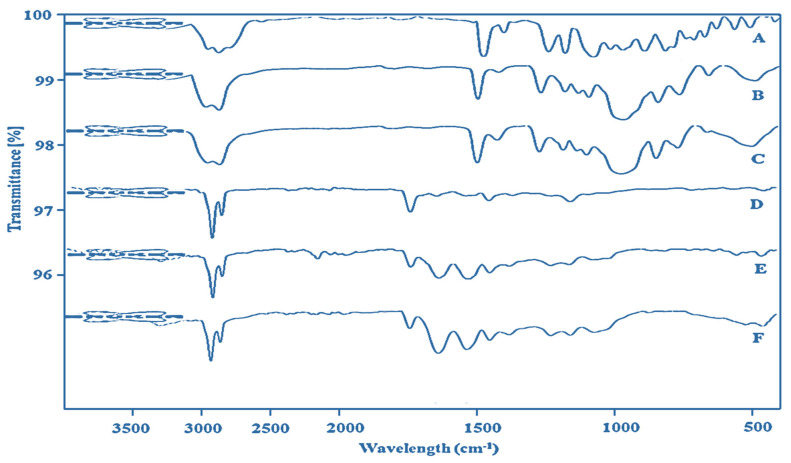
FTIR spectra: (A) Bergamot oil, (B) cremophor EL, (C) labrasol, (D), luteolin, (E) blank CNE4, and (F) luteolin loaded LCNE4.

In this study, we prepared nanoemulsions using various excipients and they were expected to be free from any chemical interactions among them. Therefore, FTIR results showed that the chemical fingerprint of the drug (LUT), excipients (BO, CEL, and LAB), and the optimized formulations (CNE4 and LCNE4) were found to be preserved as shown in Figure 4A–F. Pure BO showed characteristic C-H (3080 cm^−1^), C-N (1244 cm^−1^), and C=O (1734.92 cm^−1^) band vibrations which may be due to linalyl acetate as the major constituent present in BO as shown in Figure 4A. The characteristic observed peaks at 795.29, 922.86, 1244, and 1370 cm^−1^ indicated unsaturation (double bond as C=C) in limonene present in BO [46]. Notably, the presence of an intense band at 2934 and the stretching band of C=C at 1642 cm^−1^ in the spectra of BO of Figure 4A confirmed the valence vibration of the C-H functional group (methylene C-H band vibration) of limonene present in BO [46]. Characteristic peaks due to C=O, C=C, and O-H vibrations were observed in cremophor EL as illustrated in Figure 4B. Labrasol exhibited characteristic peaks at 2931 and 2865.54 cm^−1^ (C-H stretching), 1730.56 cm^−1^ (C-O stretching), and 1097.29 cm^−1^ (C-O stretching), as shown in Figure 4B, and these are close to reported values [47]. LUT is chemically a tetrahydroxy flavone with two aromatic rings [15]. The pure drug revealed a characteristic absorption peak at 1300–1400 cm^−1^ due to phenolic O-H bending vibration [15]. A weak stretching band (1662.0 cm^−1^) is due to C=O vibration present in the central heterocyclic ring of LUT [15]. Figure 4E shows characteristic peaks of the combined excipients present in the blank formulation. However, characteristic (but less intense) peaks of LUT were present in the optimized formulation, which may be due to the unentrapped content of the drug (Figure 4F). Thus, retained characteristic peaks present in the optimized formulation corroborated the compatibility of LUT with excipients used in the formulation.

**Figure 5:**
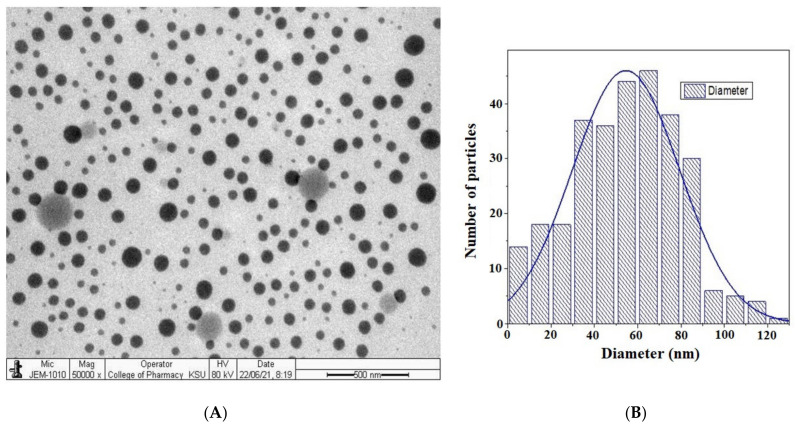
(**A**) Transmission electron microscopy (TEM) image of optimized formulation, and (**B**) corresponding histogram of particle size versus particle number.

Morphologically, the optimized formulation was spherical and well dispersed, as shown in Figure 5A, which may be due to the imposed cationic charge. The globular size histogram shows that the observed size values were less than 100 nm in the specific visualized area during TEM scanning (Figure 5B). This histogram corroborated the homogeneous nature of the dispersed globular size.

### 4.8. Drug Content

The drug content of all formulations (CNE1–CNE9), and ANE4 were estimated and they were found to be in the range of 97.9–99.7%. The result showed that there was a certain amount of drug loss due to the preparation steps and analysis procedure. However, these losses did not exceed ˃2.0%. This study suggested that the chances of drug degradation due to physical and chemical triggering factors were insignificant.

### 4.9. In Vitro Drug Release

**Figure 6:**
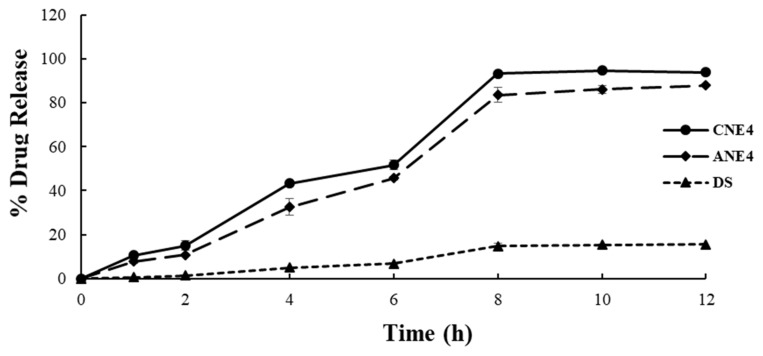
In vitro drug release (%) of LUT from various formulations (CNE4, ANE4, and DS).

The optimized formulation “CNE4” was investigated for in vitro drug release pattern in physiological buffer (PBS), and compared against anionic ANE4 and DS under similar experimental conditions. LUT is poorly soluble at physiological pH. Therefore, it was anticipated that there would be limited drug release from the suspension formulation, as observed in Figure 6. Formulated CNE4 and ANE4 nanocarriers solubilized LUT and were loaded in the lipidic phase of the nanoemulsion. A comparative release profile of these formulations is illustrated in Figure 6, wherein CNE4 and ANE4 exhibited significantly high drug release in PBS. It was clear from the release pattern that CNE4 and ANE4 demonstrated a relatively rapid release of LUT as compared to DS in PBS medium over a period of 12 h. Percent drug release values (%DR) from CNE4, ANE4, and DS were found to be 93.9 ± 0.38%, 87.84 ± 0.56%, and 15.59 ± 0.41%, respectively. Thus, they showed 6.02 and 5.63 times higher release than the drug DS after 12 h. This facilitated release of LUT from CNE4 and ANE4 may correlate with improved drug solubilization in nanoemulsion carriers. Notably, the imposed cationic charge on the globular surface of CNE4 did not impact on the in vitro drug release behavior in the same medium. Moreover, DS exhibited limited drug release, which may be due to poor solubility of LUT in saline buffer solution at pH 7.4. Percent drug release from DS was about 1.7% within the initial 2 h, which is in close agreement with reported findings (3.2%) [12]. In this study, DS was used as a control for comparison and showed no interaction with the dialysis membrane. Notably, CNE4 and ANE4 contain surfactant (CEL) and co-surfactant (LAB), which contributed to the drug solubilization when loaded in the nanoemulsion carrier and, subsequently, the release behavior [12].

### 4.10. Ex Vivo Permeation and Drug Deposition Studies

The optimized formulations (CNE4 and ANE4) and DS were intended for topical application to control breast cancer using LUT loaded nanoemulsions. Nanoemulsions primarily composed of cationic charge inducers (OA and stearylamine) are reported to potentiate drug permeation and absorption via augmented interactions with and cellular internalization in negatively charged epidermal or intestinal epithelial cells [48]. However, excessive use of a cationic charge inducer may cause irritation and toxicity. Therefore, it needs to be optimized for safe delivery. Some authors reported about 2% *v*/*v* as the recommended concentration of these charge inducers, which is higher than the concentration used in the present study (0.05% of OA) [48]. It was expected that LUT loaded in cationic nanoemulsions with a large surface area due to nanonization and imposed cationic charge may facilitate in vivo drug permeation and targeted flux. This may improve the therapeutic efficacy of LUT to control breast cancer if treated topically. Moreover, this approach can be advantageous compared to oral treatments and other routes of administration by avoiding gastric-triggered instability and limited oral absorption and providing targeted delivery to the tumor lesion (if delivered topically) and high patient compliance. Lubna et al. investigated improved skin permeation of LUT loaded vesicular systems to control inflammation caused by arthritis and they achieved ~93.0 µg/cm^2^/h as permeation flux and 2.66 as the enhancement ratio as compared to the drug suspension (control) on rat skin [11]. They explained the improved permeation as being due to structural medication in the stratum corneum through niosomes. In this study, we hypothesized that augmented permeation of LUT would occur across the stratum corneum of rat skin, using a combination of permeation mechanisms working together and imposed cationic charge for electrostatic interaction with a negatively charged cell surface, increased surface area using a nanoemulsion able to permeate across tiny skin pores and through the follicular route, S_mix_ components able to cause reversible structural changes in the stratum corneum, and improved LUT solubilization in BO of the nanoemulsion [12]. Ansari et al. reported 3-fold higher permeation of LUT loaded in a self-nanoemulsifying drug delivery system (SNEDDS) in rats [12]. BO is an essential oil of the generally regarded as safe (GRAS) category and is reported to have more cytotoxic potential when formulated in nanoemulsions [49]. Thus, improved permeation of LUT loaded cationic nanogobules may be detrimental to cancerous cells.

**Figure 7:**
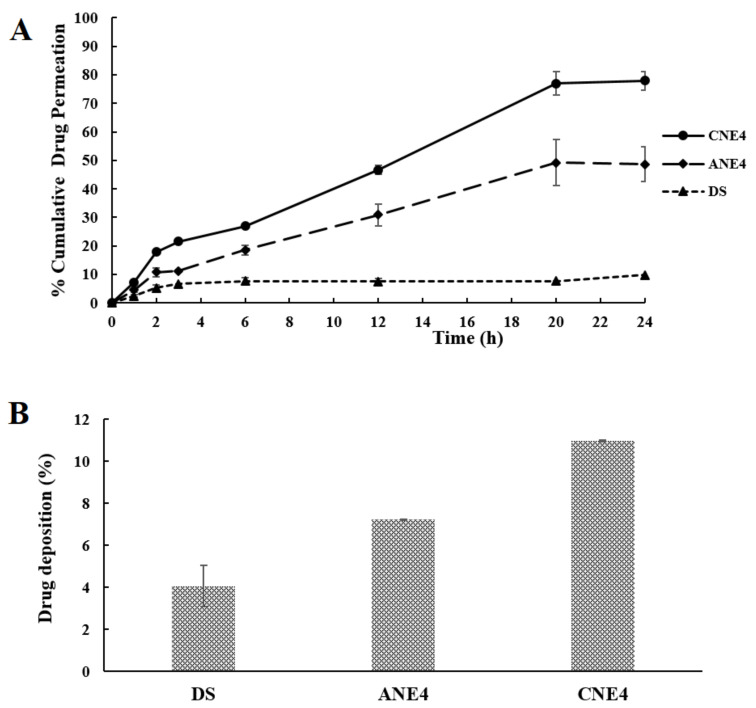
(**A**) Ex vivo permeation (% cumulative drug permeated per cm^2^) study of CNE4, ANE4, and DS for period of 24 h using rat skin, and (**B**) drug deposition (% DD/cm^2^) of CNE4, ANE4, and DS for period of 24 h using rat skin.

Results of ex vivo permeation (a comparative graph) are presented in Figure 7A. Percentages of cumulative drug permeated across rat skin were 77.96%, 48.59%, and 9.74% for CNE4, ANE4, and DS, respectively. Permeation flux values of CNE4, ANE4, and DS were obtained as 138.82, 86.53, and 16.86 µg/cm^2^/h, respectively (Table 3). Thus, the value of permeation flux (86.53 µg/cm^2^/h) was in close agreement with the reported value (93.0 µg/cm^2^/h) achieved in SNEDDSs [12]. However, the permeation flux value of CNE4 was found to be significantly high as compared to ANE4, DS, and the reported value (93.0 µg/cm^2^/h). Comparing these values, the flux achieved through CNE4 was 8.23- and 1.6-fold higher than DS and ANE4, respectively. Despite the similar composition, CNE4 exhibited relatively higher permeation flux as compared to ANE4, which may be due to the imposed cationic charge responsible for maximized internalization with a negatively charged cellular surface [30]. The calculated enhancement ratios obtained from CNE4 and ANE4 were 8.23 and 5.13, respectively. The permeation flux values of CNE4 and ANE4 were 1.92- and 1.24-fold higher than the roughly estimated targeted flux in the human body (69.92 µg/cm^2^/h). Thus, this finding suggested that the explored CNE4 and ANE4 can efficiently deliver LUT with targeted flux for high therapeutic efficacy if applied topically/transdermally. However, the flux value from DS was lower than the estimated targeted flux and, therefore, DS cannot produce therapeutic efficacy (applied transdermally). It is a well-established fact that the prominent SC layer impedes permeation of insoluble LUT and other exogenous compounds due to flattened corneocytes cemented with ceramides (skin lipoprotein) [50].

In the literature, topical application of a permeant may follow three possible pathways: (a) intercellular route, (b) transcellular routes, and (c) appendageal routes (hair follicles, sebaceous glands, and sweat ducts) (~0.1% fractional appendage area available for permeation). The intercellular and transcellular routes constitute the prime routes of permeation and a together known as “transepidermal pathways” [51]. It is notable that “intercellular route” is the preferred route for insoluble drug candidates, such as LUT, rifampicin, and molecules with a high molecular weight [51]. Nanoemulsion offers improved permeation and drug deposition by structural changes in the lipophilic pathway by reversible transformation of the SC [52]. Furthermore, the diffusion of LUT across the SC may be the result of lateral diffusion and intramembrane transbilayer transport [53]. Application of a nanoemulsion carrier can make it possible to permeate LUT via the hair follicles as these nanocarriers can easily diffuse along this type of shunt route [18]. Thus, cationic nanoemulsions may be promoted through these shunt routes as the main pathway of LUT permeation [18]. The result of the percentage of drug deposition (%DD/cm^2^) is presented in Figure 7B and Table 3. Drug deposited in the skin was 10.98%, 7.23%, and 4.06% for CNE4, ANE4, and DS, respectively, after 24 h. Thus, drug deposition was found to be higher with CNE4 and ANE4 as compared to DS. This may be due to cationic nanoemulsion-mediated enhanced permeation and electrostatic interaction with the cellular surface. DS showed limited drug deposition (%DD) and permeation flux due to the lipophilic nature of LUT and crystalline hydrophobic SC layer of the skin. Thus, imposed electrostatic interaction, nanonization, and surfactant-mediated reversible structural changes worked collectively to enhance LUT permeation flux, enhancement ratio, and drug deposition for targeted therapeutic efficacy.

## 5. Conclusions

LUT is a natural flavonoid possessing anticancer activity and several other therapeutic benefits. Naturally obtained BO, CEL, and labrasol were explored to fabricate cationic nanoemulsions to achieve the desired size, zeta potential, stability, percentage of transmittance, in vitro drug release, and ex vivo permeation parameters. The results showed that cationic and anionic nanoemulsions showed insignificant differences in % drug release which may be due to the efficient emulsification of the developed nanoemulsion in PBS medium. Moreover, the imposed cationic charge could not interact with the membrane during the release process. However, the percentage of cumulative permeation, steady state permeation flux, enhancement ratio, and DD values were remarkably improved in CNE4 and ANE4 as compared to DS. Moreover, the imposed cationic charge on CNE4 significantly enhanced permeation parameters as compared to ANE4, suggesting efficient internalization and interaction of nanoglobules with the skin cell surface through electrostatic interaction. Therefore, CNE4 may be synergistically more able to control breast cancer if loaded with luteolin. Thus, naturally obtained LUT and BO may be a promising approach to formulate cationic nanoemulsions for enhanced transdermal delivery.

## Figures and Tables

**Table 1:** Composition and evaluation parameters of selected luteolin loaded cationic nanoemulsions containing constant amount of oleylamine (0.05% *w*/*v*) as cationic charge inducer.

Code	OA (% *w*/*w*)	BO (% *w*/*w*)	S_mix_^†^ Ratio (CEL */L ^Φ^)	Aqueous (% *w*/*w*)	Mean Droplet Size (nm)	PDI	ZP (mV)	pH	TDC (%)
CNE1	0.5	9.5	1:1	67	373.2 ± 11.4	0.46	+17.1	7.4	97.9
CNE2	0.5	11.5	1:2	60	263.6 ± 9.7	0.47	+14.6	7.5	98.2
CNE3	0.5	17.0	1:3	50	158.7 ± 8.9	0.18	+15.0	7.9	98.5
CNE4	0.5	14.5	2:1	57	110.6 ± 8.1	0.15	+26.1	7.4	99.7
CNE5	0.5	18.0	3:1	45	307.0 ± 11.2	0.91	+34.1	7.4	97.9
CNE6	0.5	16.5	1:4	37	321.7 ± 12.1	0.69	+17.0	7.5	99.3
CNE7	0.5	20.5	4:1	35	407.5 ± 13.6	0.65	+35.0	7.8	98.5
CNE8	0.5	17.5	2:3	55	254.7 ± 10.4	0.57	+30.0	7.8	99.1
CNE9	0.5	17.0	3:2	65	174.6 ± 7.8	0.82	+39.0	7.8	99.7
ANE4	0.0	15.0	2:1	57	134.4 ± 9.5	0.171	−28.9	7.5	98.3

Value represented as mean ± SD (*n* = 3), * CEL = Cremophor EL as surfactant across the skin, ^†^ S_mix_ = Surfactant:co-surfactant ratio, ^Φ^ L = Cremophor EL as surfactant and labrasol as co-surfactant, S_mix_ = C: L; BO = Bergamot oil; OA = Oleylamine, DMSO = Dimethyl sulfoxide; ANE4: Anionic NE4. LUT (3.0% *w*/*w*) previously dissolved in 10% *w*/*w* of DMSO before adding to organic phase.

**Table 2:** Thermodynamic stability testing of developed cationic nanoemulsions loaded with luteolin (series of heating and cooling cycles).

Code	H/C	Centrifugation	Freezing Temperature	Inference
CNE1	✓	✓	✓	pass
CNE2	✓	✓	✓	pass
CNE3	✓	✓	✓	pass
CNE4	✓	✓	✓	pass
CNE5	✓	✓	✓	pass
CNE6	×	×	×	fail
CNE7	✓	✓	✓	pass
CNE8	×	×	×	fail
CNE9	✓	✓	✓	pass
ANE4	✓	✓	✓	pass

Note: H/C = Heating and subsequent cooling temperature; ✓ = Formulation returned to original form; × = Formulation was unstable due to visually observed signs of precipitation or phase separation.

**Table 3:** Ex vivo permeation parameters of luteolin loaded cationic nanoemulsion after 24 h of study.

Code	Permeation at 24 h (%/cm^2^)	*f* (µg/cm^2^ h)	DD (%/cm^2^)	ER^2^
CNE4	77.96 ± 3.5	138.82 ± 8.4	10.98 ± 0.33	8.23
ANE4	44.59 ± 1.7	86.53 ± 2.7	7.23 ± 0.12	5.13
Drug suspension (DS)	9.74 ± 0.6	16.86 ± 0.95	4.06 ± 0.05	-

Value represented as mean ± SD (*n* = 3), ER^2^ = Enhancement ratio, DD = Drug deposition, f = Permeation flux.

## Data Availability

Not applicable.
